# Charge Carrier Localization in Doped Perovskite Nanocrystals Enhances Radiative Recombination

**DOI:** 10.1021/jacs.1c01567

**Published:** 2021-05-16

**Authors:** Sascha Feldmann, Mahesh K. Gangishetty, Ivona Bravić, Timo Neumann, Bo Peng, Thomas Winkler, Richard H. Friend, Bartomeu Monserrat, Daniel N. Congreve, Felix Deschler

**Affiliations:** † Cavendish Laboratory, University of Cambridge, Cambridge CB30HE, U.K.; ‡ Rowland Institute, Harvard University, Cambridge, Massachusetts 02142, United States; § Department of Chemistry and Physics, Mississippi State University, Mississippi State, Mississippi 39762, United States; ∥ Walter Schottky Institute, Technical University of Munich, Garching 85748, Germany; ⊥ Department of Materials Science and Metallurgy, University of Cambridge, Cambridge CB30FS, U.K.

## Abstract

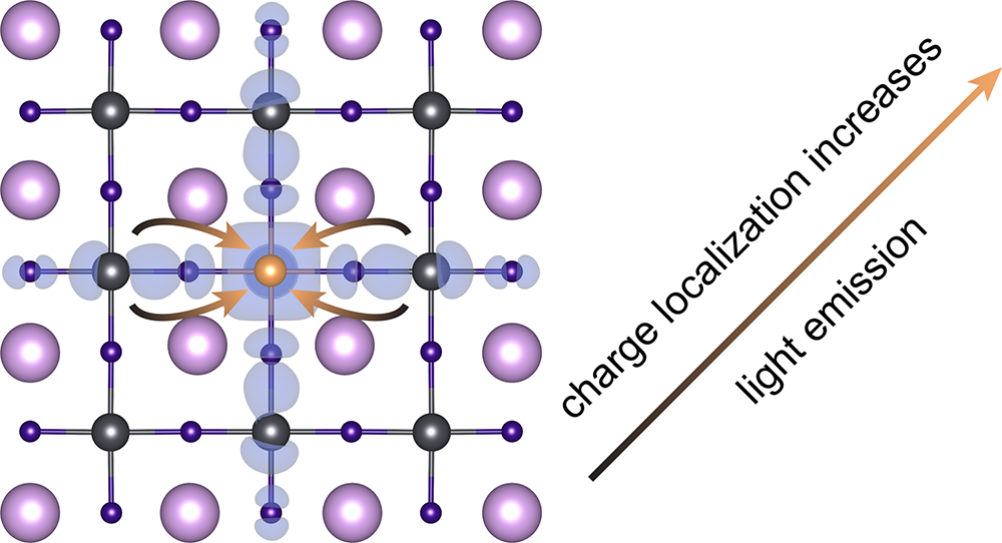

Nanocrystals based on halide perovskites offer a promising material platform for highly efficient lighting. Using transient optical spectroscopy, we study excitation recombination dynamics in manganese-doped CsPb(Cl,Br)_3_ perovskite nanocrystals. We find an increase in the intrinsic excitonic radiative recombination rate upon doping, which is typically a challenging material property to tailor. Supported by *ab initio* calculations, we can attribute the enhanced emission rates to increased charge carrier localization through lattice periodicity breaking from Mn dopants, which increases the overlap of electron and hole wave functions locally and thus the oscillator strength of excitons in their vicinity. Our report of a fundamental strategy for improving luminescence efficiencies in perovskite nanocrystals will be valuable for maximizing efficiencies in light-emitting applications.

## Introduction

Metal-halide perovskite nanocrystals (NCs) show high brightness, spectral tunability, and excellent color gamut, making them ideal candidates for low-cost and highly efficient light-emitting diodes (LEDs).^1−3^ Recently, doping of these NCs with manganese ions has increased efficiencies in perovskite-based LEDs.^4^

Doping of II–VI, II–V, and group-IV nanocrystal semiconductors was shown to successfully modify electronic, optical, and magnetic properties^5−8^ based on increased conductivity through n-/p-type doping or increased interactions between carriers and spins of magnetic dopants due to the confinement within the NC. So far, efforts to utilize dopants for exciton localization have been limited to protecting materials against photooxidation by suppressing degradation reactions at the NC surface under prolonged illumination, e*.*g., in solar cells.^9^ Yet, in these systems, efficient energy transfer to the dopant results in complete quenching of the host exciton emission. Alternatively, attempts to exploit exciton localization to increase emission rates rely on core–shell architectures for wave function engineering,^10,11^ which generally involve multistep synthesis.^12−14^

We now report in this article, combining optical spectroscopy and *ab initio* calculations, that manganese doping in perovskite NCs increases the radiative exciton recombination rate, which we demonstrate to arise from increased overlap of electron and hole wave functions from dopant-induced exciton localization. Our results identify a fundamental mechanism for controlling band structure and excitonic properties of metal-halide perovskite nanocrystals, which provides a promising concept for further improving high-performance LEDs.

## Results and Discussion

**Figure 1:**
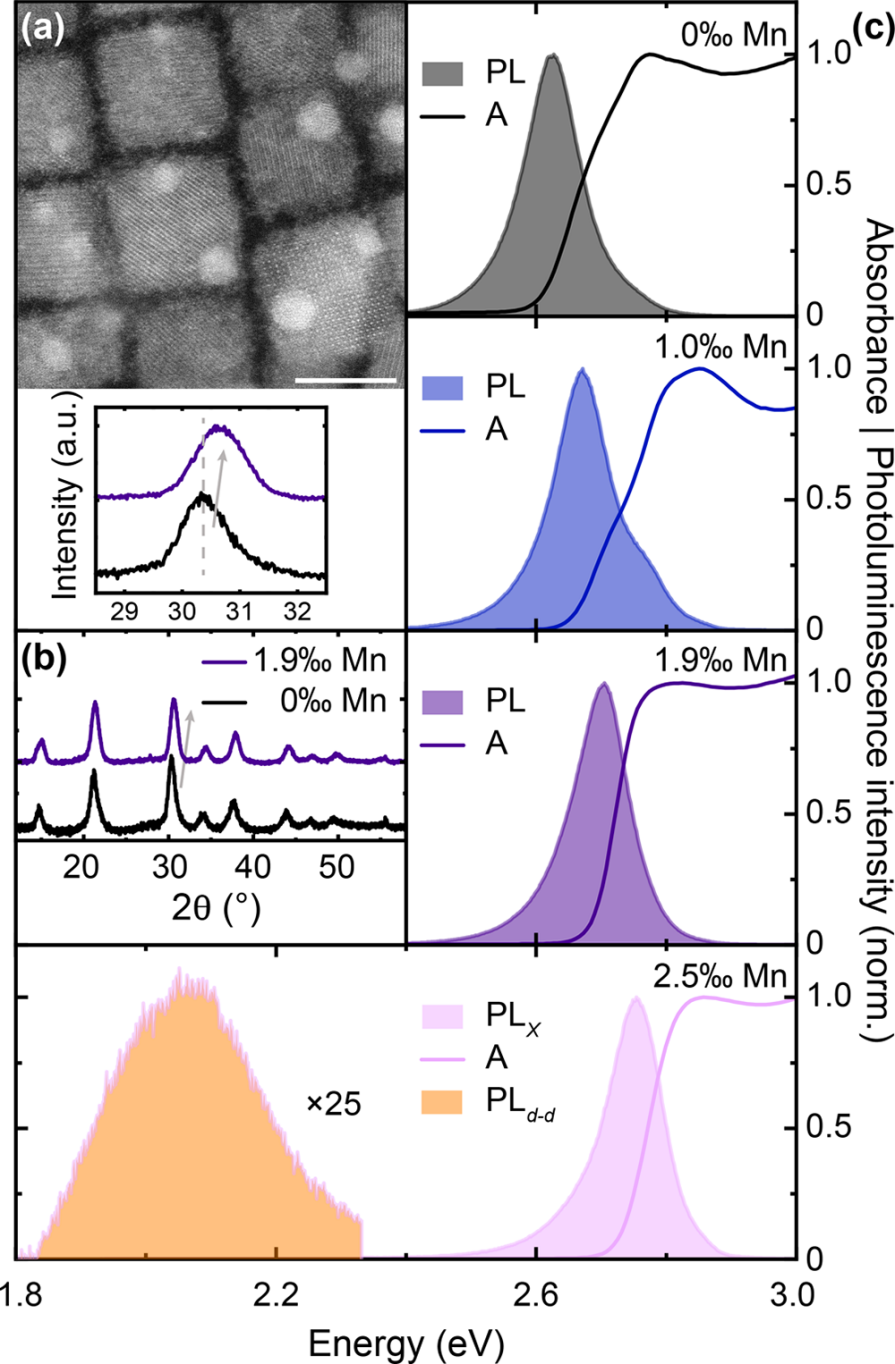
Structural and optical properties of manganese-doped perovskite nanocrystals. (a) Transmission electron microscopy image of doped NCs (shown exemplarily for 1.9 atomic ‰ Mn:Pb) with cubic morphology and an average crystal size of 12 ± 2 nm. Scale bar is 10 nm. (b) X-ray diffractogram of undoped (black) and 1.9‰-Mn-doped (purple) nanocrystals, respectively, showing slight lattice contraction upon doping. (c) Steady-state absorbance (bold lines) and photoluminescence (PL, filled) of NC solutions for increasing Mn-doping, showing a doping-induced blue-shift of the excitonic transition around 2.7 eV. Samples were photoexcited with 3.1 eV pulsed excitation at a fluence of 127 μW cm^–2^ (see Figure S1 for full-range spectra).

We first characterize the synthesized Mn-doped CsPb(Cl,Br)_3_ perovskite NCs with regard to their structural and optical properties (see Supporting Information for synthesis protocols^4^). Transmission electron microscopy (TEM) confirms a cubic structure of the NCs with an average size of approximately 12 nm [Figure 1(a)].

Similar sizes are found for all undoped and manganese-doped crystals studied here. This allows us to compare the impact of Mn-doping on optoelectronic properties quantitatively, excluding effects related to dielectric screening and quantum confinement that might arise from different crystal shapes or sizes.^15−17^ We further take X-ray diffraction data [Figure 1(b)] on undoped NC films and those doped with a 1.9‰ Mn:Pb atomic ratio, as determined by inductively coupled plasma mass spectrometry. We find very similar diffraction peak patterns across all compositions, with peaks shifting to higher angles with increasing Mn-doping, as reported.^18−21^ This indicates a moderate degree of lattice contraction, which is expected from the smaller manganese(II) ion (1.4 Å) substituting the larger lead(II) ion (1.8 Å) in the octahedral halide coordination sphere. In the steady-state absorption and photoluminescence (PL) spectra [Figure 1(c)] we observe a gradual blue-shift in absorption and emission with increasing doping level from 0 to 2.5‰, consistent with previous reports.^19,20,22^ Notably, while all samples exhibit an intense blue excitonic emission (*X*) at around 2.7 eV, only the most doped 2.5‰ sample shows the well-known spin-forbidden manganese(II) ^4^*T*_1_ → ^6^*A*_1_ (d–d) transition centered at 2.1 eV with long emission lifetimes (see Figure S2). Most notably, the excitonic emission intensity increases by a factor of 3.3 from 0 to 1.9‰ manganese doping and then drops again for the 2.5‰ sample (see also Figure S1). We find similar increases upon doping for the absorption cross-section, up to a factor of 3.0 ± 0.4, which we derived from transient absorption (discussed in detail further below), a quantity that is generally challenging to extract reliably from linear absorption measurements. In the following we will investigate the fundamental origin of this advantageous excitonic emission increase.

**Figure 2:**
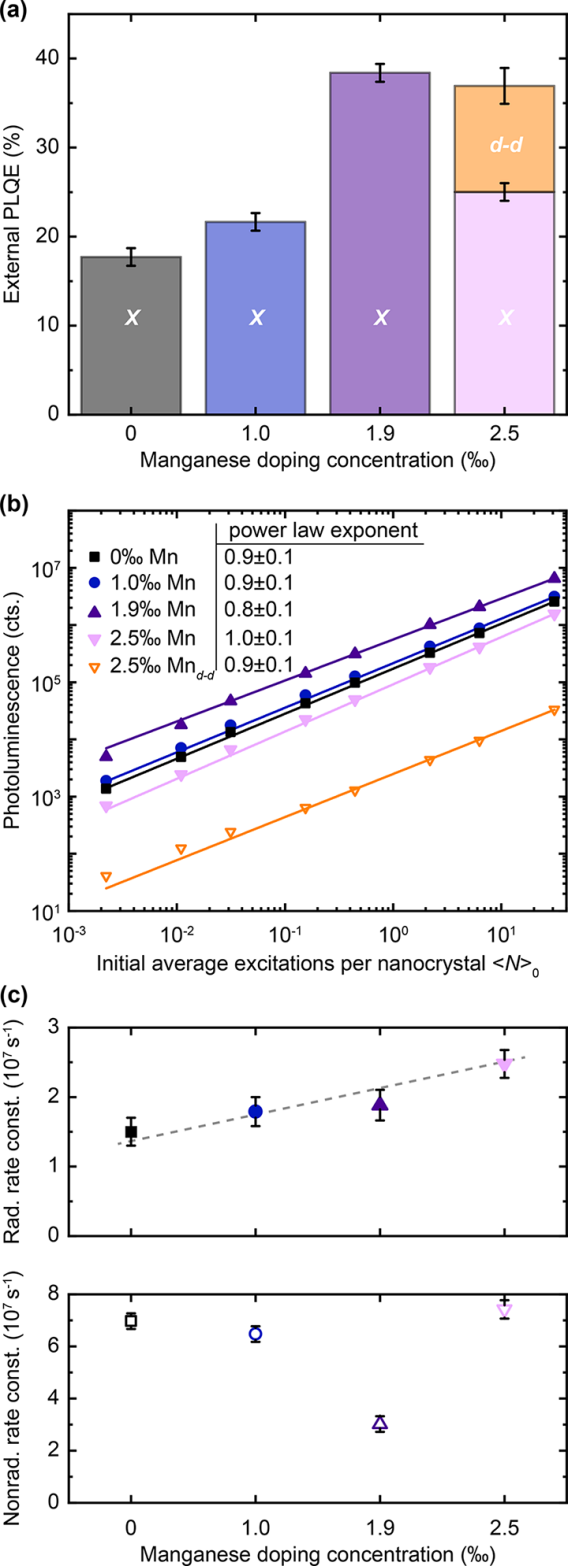
Impact of manganese doping on radiative recombination. (a) Photoluminescence quantum efficiency (PLQE) as a function of manganese doping for the perovskite excitonic (*X*) and Mn (d–d) emission. (b) Fluence dependence of spectrally integrated PL intensity of exciton and Mn emission, respectively. Solid lines are power-law fits to *P*^*m*^ with excitation power *P* and slope *m* indicated in the figure panel. (c) Radiative and nonradiative recombination rates on Mn doping, showing a continuous increase in radiative rate and a minimum in nonradiative rate at 1.9‰ Mn doping. Gray dashed line is a guide to the eye. Samples were photoexcited with 3.1 eV pulses at a repetition rate of 1 kHz (pulse duration ∼100 fs).

In Figure 2, we quantify the positive impact of the Mn-doping on the emission properties.

We find that the photoluminescence quantum efficiency (PLQE) of the excitonic emission increases by a factor of 2.2 up to 1.9‰ manganese doping, reaching values around 40% [Figure 2(a)]. For the highest studied doping level, the PLQE of the excitonic emission decreases, while the Mn d–d emission becomes detectable. This suggests the existence of a threshold doping level between 1.9 and 2.5‰, upon which energy transfer from the perovskite host exciton to the dopant becomes significant. The fact that the PLQE gains do not fully reflect the PL intensity increases (up to 3.3-fold) indicates an increase in absorption, which we will rationalize further below from a doping-induced increase in oscillator strength from localization effects.

Next, we investigate the fluence dependence of the luminescence [Figure 2(b)]. We find that the photoluminescence of all compositions scales linearly over the studied range of excitation fluences. This indicates that the luminescence is dominated by exciton recombination over all studied fluences.^15^ The excitonic behavior agrees with the pronounced excitonic absorption peak and weak quantum confinement^15^ of excitons with a predicted Bohr radius of about 2 nm in our NCs with a size of about 12 nm. With a unit cell volume of ∼200 Å^3^ (in agreement from density functional theory (DFT)- and X-ray diffraction (XRD)-based results) and an NC volume of ∼8 × 10^–24^ m^3^ from our STEM measurements, we calculate an average density of ∼40–100 Mn dopants per NC for a doping concentration of 1–2.5‰. For cube-shaped nanocrystals, the mean spacing between Mn dopants is thus ∼6–4 nm for the given range of doping concentrations. In contrast, the exciton diffusion lengths reported for cesium lead halide nanocrystals vary between 30 and 200 nm.^23^ Thus, photoexcited excitons will experience the presence of at least one manganese dopant, and we will discuss the nature and consequences of these interactions later on.

From time-resolved single-photon counting experiments (TCSPC, Figure S2) we further find that the 1.9‰ composition exhibits the longest average PL lifetime. Combining PLQE and TCPSC results, we readily quantify the radiative and nonradiative recombination rates [Figure 2(c), see Supporting Information for details of calculations]. The nonradiative rate, which represents luminescence loss channels, decreases upon Mn-doping, reaching its minimum for the composition with the highest PLQE. This initial decrease in nonradiative rate we observe at low doping concentrations is likely a consequence of the added MnCl_2_ filling the pre-existing halide vacancies, thus reducing the trap density and with it the nonradiative rate. We assign the observed minimum in nonradiative rate at 1.9‰ Mn doping concentration to the limit after which Mn cluster formation takes place.^24^ This argument is further supported by the onset of the observable d–d emission at this concentration, for which the Mn d orbitals in those clusters start to hybridize and form the observable new energy band. Unexpectedly, we further find that the radiative recombination rate increases with doping level, leading to the highest radiative rate of 2.5 × 10^7^ s^–1^ for the 2.5‰ doping level.

**Figure 3:**
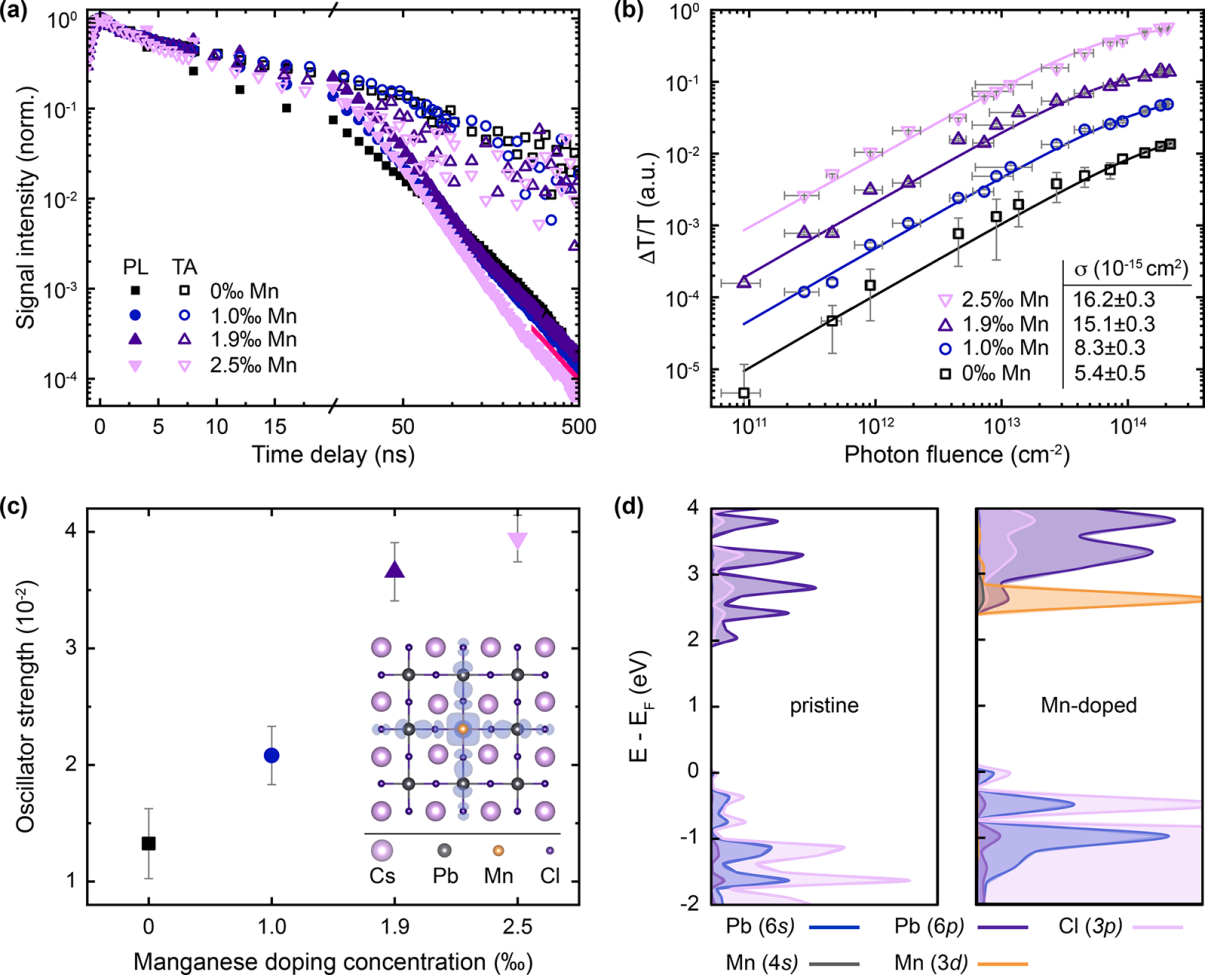
Excitation dynamics and localization in manganese-doped perovskite nanocrystals. (a) Transient photoluminescence (PL) and transient absorption (TA) ground-state bleach (GSB) kinetics. Monoexponential decay dominates at time delays beyond ∼200 ns for all compositions with a shared lifetime of 116 ± 2 ns (red line). PL and TA signals were spectrally integrated over the respective peak maximum; initial average density approximately 1.1 excitations per NC. (b) GSB signal within the nanosecond-resolution of the experiment as a function of incident photon fluence. The absorption cross-section values σ extracted from fits (solid lines) increase with doping level. All samples excited at 3.1 eV for (a) and (b). (c) Oscillator strength per unit cell of the band-to-band transitions determined from experimental absorption cross-sections. Inset: First-principles calculation of the electron density for Mn-doped perovskite, showing carrier localization at the manganese dopant. Value at the isosurface is 2.36 × 10^–3^ e Å^–3^. (d) Projected density of states for CsPbCl_3_ (left) and CsPb_0.963_Mn_0.037_Cl_3_ (right). We find hybridization of the Mn 4s states with the host, which leads to charge localization responsible for the observed oscillator strength increases.

This increase in the radiative recombination rate, which is an intrinsic material property, indicates that the manganese dopants fundamentally alter electronic structure and carrier dynamics in the perovskite host. While the reduced nonradiative rate observed here and reported before^4,25^ relies on optimization strategies based on trap passivation,^26^ it is the increase in radiative rate through doping that represents a fundamentally novel finding for our systems. Even though the PLQE gains from nonradiative rate reduction are quantitatively larger in our systems than the gains from the radiative rate increases, it is the latter observation that presents a powerful result for the materials’ performance, since the ability to enhance radiative rates improves the performance even for materials that have reached zero nonradiative losses, for example in lasing and quantum emission applications.

To establish the origin of the increased radiative recombination rates, we track the excitation dynamics by comparing the kinetics of the excitonic PL and the ground-state bleach (GSB) from transient absorption (TA) [Figure 3(a); see Figure S3 for full TA map].

TA and PL both probe the decay of photogenerated excitons. TA signals probe how many excitations remain after a certain time delay, including those whose kinetics are dominated by nonradiative decay and, thus, will not contribute to PL strongly. The PL signal traces only the fraction of radiatively recombining excitons per unit time. Thus, identical kinetics for TA and PL signals are indicative for radiative exciton recombination. A divergence of the kinetics with a faster PL decay than TA decay indicates nonradiative trapping of either electrons or holes instead. This is because removal of one excited charge carrier type results in the immediate loss of an exciton that could recombine radiatively and contribute to the PL signal, whereas the leftover electron or hole in either band-edge can still partially contribute to the TA signal.^17,18,27^

We find that within the initial few nanoseconds the PL matches closely the TA signal, hence indicating predominantly radiative recombination. From about 5 ns onwards, for the undoped sample the PL kinetics then begin to diverge from the TA kinetics and decay faster, thus indicating nonradiative losses from electron or hole trapping. Upon doping, the fraction of radiative exciton decay increases, as indicated by the PLQE measurements. Therefore, as expected, we observe that the divergence of PL and TA kinetics shifts to later time delays (of about 15 ns) upon doping.

The longer PL lifetimes upon doping indicate a reduction in nonradiative decay. TA kinetics decay faster for the doped samples up to 50 ns, which is unexpected for reduced nonradiative recombination rates and a further clear indication for an increase in radiative rates. For the longer time delays beyond approximately 200 ns all compositions follow a monoexponential decay with similar lifetimes (Figure S3). We assign this long-lived component to nonradiative trap-assisted recombination.^28,29^

In order to identify the fundamental mechanism enabling the radiative rate enhancement, we determine the oscillator strengths from the absorption cross-sections. We first calculate the average number of excitations per NC ⟨*N*⟩ (see Supporting Information for details on calculations), which is directly related to the absorption cross-section σ via ⟨*N*⟩ = σ*j*, with the photon fluence *j* (number of photons incident per cm^2^), following established approaches.^17,30−32^ After multiexciton recombination is completed, which we confirm to be the case in our experiments by extracting biexciton lifetimes of 10–25 ps (see Figure S5 and ref (18)), each NC can at most be occupied with one exciton. Hence, when plotting the GSB intensity (after initial cooling and biexciton decay) as a function of photon fluence, we observe a saturation behavior at high fluences [Figure 3(b)]. With the probability *p* of an NC to contain *i* excitations , this fluence dependence can be described by a Poisson distribution with the GSB intensity being proportional to 1 – *p*_0_ = 1 – e^–⟨*N*⟩^.^30,33^ With this, we extract values for the absorption cross-section from our TA data, which we find to increase with Mn-doping level, yielding a nearly 3-fold increase in the σ value for the highest doping concentration. With the measured absorption cross-section, we can calculate the oscillator strength *f* for the band-to-band transition [Figure 3(c)] employing a modified version of the Strickler–Berg relation:^34^
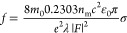
1where *m*_0_ is the free electron rest mass, *n*_m_ the refractive index of the surrounding medium, here toluene, *c* the speed of light in a vacuum, ε_0_ the vacuum permittivity, *e* the elementary charge, and λ the wavelength of the optical transition. *F* = 3ε_m_/(ε_s_ + 2ε_m_) is the local field factor to account for the screening of the nanoparticle modeled as a sphere, with ε_m_ and ε_s_ the dielectric constants of the medium and semiconductor, respectively. Since the oscillator strength quantity originates from a single-oscillator model, the values obtained were divided by the ratio of unit cell volume to NC volume before plotting, though the sum following the Thomas–Reiche–Kuhn rule could also be used.^35,36^ We find a 3-fold increase in the oscillator strength for the most doped sample. We find very similar values for the oscillator strength from an analysis of our radiative rates, thus confirming our finding from two different sets of experiments, fundamentally connected via the Einstein relations.^37^ The increase in oscillator strength, which is a direct measure of the dipole matrix element of the transition, can be explained by an increase of the electron–hole overlap Θ_e-h_, which is given by^38^
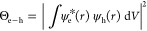
2where ψ_e__,h_(*r*) are the electron and hole wave functions, respectively, integrated over the volume *V*. A second potential explanation for changes in the oscillator strength changes is changes in the dielectric constant of the material, which will not be relevant in our permille doping regime where 60–80 Mn atoms are added to the ∼5 × 10^5^ atoms per nanocrystal. We find Θ_e-h_ to increase by 158% for the highest doping level (see Supporting Information and refs (38) and (39) for details on the calculation). Thus, we propose that the local distortion of the perovskite lattice induced by the Mn dopants is likely to break the lattice periodicity in the interior of the NC, which increases the electron–hole overlap of excitons in the vicinity of the dopant and thus their probability of radiative decay. Hence, the modification of the electronic structure induced by the dopants occurs in the excited state, altering the recombination of excitons after photoexcitation, and could thus be captured via our transient absorption and PL experiments. Such localization also reduces the likelihood of nonradiative recombination through diffusion of carriers to trap sites and could provide an additional explanation for the observed reduction in the nonradiative recombination rate upon doping.

To further elucidate the influence of Mn-doping on exciton recombination, we perform electronic structure calculations using DFT (see Supporting Information for details). Via the supercell approach, we model pristine and Mn-doped CsPbX_3_ compositions for X = Cl, Br, or a mixture thereof. We confirm a direct band gap at the *R*-point of the Brillouin zone, which increases by ∼0.2 eV upon doping, in agreement with our absorption results. We find that, once the Pb is partially replaced with Mn, the valence band maximum (VBM) is perturbed, resulting in a band that mostly resembles the energetically lower lying isolated halide p orbitals. Further, the perturbation in the periodicity of the Pb 6p orbitals leads to the destabilization of the conduction band minimum (CBM). Both perturbations hence contribute toward the observed widening of the band gap. Importantly, these perturbations also reduce the dispersion of both VBM and CBM, indicating a more localized electron and hole state with higher effective masses in the doped case (see Supporting Table T2).

We further calculate the real-space charge distribution of the CBM for pristine and Mn-doped CsPbCl_3_ using the PBEsol functional with 20% additional Hartree–Fock exchange [Figure 3(c), inset]. We observe that the charge distribution shows a more localized character compared to the undoped case (Figures S8 and S9) with the largest coefficients in proximity to the central Mn atom and along the Mn–Cl–Pb bonds, while the contributions from the remaining perovskite scaffold become negligible. This charge localization leads to an increased electron–hole wave function overlap and thus rationalizes our observed increase in radiative recombination rate^40^ upon doping, as from the above considerations, on average, every exciton will pass by such a doping cite within its lifetime. The detailed origin of this localization effect is found in the projected density of states [pDOS, Figure 3(d)]. Here, the Mn 4s states as well as the Mn 3d states energetically coincide with the CBM of the perovskite, but intriguingly, while the 3d orbitals have a negligible effect on the band edge, the 4s orbitals strongly hybridize with it and thus modify the host wave function significantly. This hybridization of the Mn 4s states with the host leads to charge localization, while the Mn 3d states do not mix with the CBM and hence form a competing decay channel at higher concentrations.

In summary, we find a structural and an electronic contribution to the effect of doping on the localization of the wave function that constitute the band edges of the perovskite. The structural effect is the lattice-periodicity breaking, which is generally caused by any *B*-site substitution and is therefore not metal-specific. This leads to less dispersive band edges compared to the undoped case and hence more localization. The electronic effect, which turns out to be stronger for Mn specifically, is the mixing of the dopant with the perovskite states that constitute the band edges. It turns out that an important prerequisite for hybridization is the relative position of the empty Mn s states and the CBM. Our calculations show that the band alignment depends on the electronic structure of both the dopant and the chosen halide (see Figure S7). With increasing chloride (and decreasing bromide) content the Mn s state and the conduction band edge come closer to one another in energy, such that hybridization, and thus localization, becomes possible. Therefore, it will be important to consider the choice of the metal dopant and the halide to exploit the electronic-induced localization effect and hence maximize the radiative rate.

Lastly, we note that the use of other transition metals as dopants could be a promising avenue to tackle the challenges mentioned above: Promising dopants could include for example nickel, where no d states should occur within the perovskite band gap, or zinc with its closed d shell. By tuning the element-dependent degree of s orbital hybridization and mitigating the formation of d states, the localization effect we observe can be exploited to maximize radiative rates.

## Conclusions

We find that manganese-doping of perovskite nanocrystals increases their luminescence yields due to an unexpected enhancement of radiative recombination rates, in combination with the reduction of nonradiative rates discussed in the literature previously. We identify the origin of the enhanced luminescence rates as an increase of the oscillator strength from stronger charge localization at doping sites, where the larger overlap of electron and hole wave functions enhances the radiative recombination of excitons in their vicinity. We stress that this localization effect provides a pathway to improve radiative rates even for materials, which may have already reached low nonradiative losses from minimized trap densities through advanced fabrication methods. Our results demonstrate how transition-metal doping provides precise control of electronic structure and exciton dynamics in metal-halide perovskite nanocrystals and how this opens a route toward very efficient light-emitting devices.

## Supporting Information Available

Methods and experimental details, additional data, and discussion (PDF)
