# Supplementary material for: Charge Carrier Localization in Doped Perovskite Nanocrystals Enhances Radiative Recombination
Source: J Am Chem Soc. 2021 May 16;143(23):8647–53. doi: 10.1021/jacs.1c01567 (PMC8297723; doi:10.1021/jacs.1c01567)
Supplement: Supplementary file 1 — ja1c01567_si_001.pdf [file ja1c01567_si_001.pdf]

Supporting Information
for
Charge carrier localization in doped perovskite nanocrystals enhances radiative recombination

Sascha Feldmann,[1]* Mahesh K. Gangishetty,[2,3] Ivona Bravić,[1] Timo Neumann,[1,4] Bo Peng,[1] Thomas Winkler,[1] Richard H. Friend,[1] Bartomeu Monserrat,[1,5] Daniel N. Congreve,[2] and Felix Deschler[1,4]*

[1] *Cavendish Laboratory, University of Cambridge, Cambridge, CB30HE, UK*

[2] *Rowland Institute, Harvard University, Cambridge, Massachusetts, 02142, USA*

[3] *Department of Chemistry and Physics, Mississippi State University, Mississippi, 39762, USA*

[4] *Walter Schottky Institute, Technical University of Munich, Garching, 85748, Germany*

[5] *Department of Materials Science and Metallurgy, University of Cambridge, Cambridge, CB30FS, UK*

*e-mail: sf561@cam.ac.uk, felix.deschler@tum.de

Methods

Nanocrystal synthesis

The detailed synthesis of undoped and Mn-doped perovskite nanocrystals can be found in our previous study [1] and the literature cited therein. Briefly, for undoped $CsPb(Cl,Br)_3$ nanocrystals, 165 mg of $PbBr_2$ (0.450 mmol, purity 98%), 83.6 mg of $PbCl_2$ (0.301 mmol, purity 98%), 20 mL of octadecene, 2 mL of oleylamine (purity 98%), 2 mL of oleic acid, and 2 mL of trioctylphosphine (purity 97%) were loaded into a 100-mL three-neck flask, dried under vacuum at 130°C for 45 min, and heated to 150°C under stirring. The yielded clear solution was heated to 165°C under N_2 protection, after which 1.7 mL of pre-heated Cs-oleate precursors was swiftly injected into the solution. After reacting for 10 s, the product was cooled to room temperature in an ice/water bath. Synthesis of low Mn^{2+}-doped perovskite nanocrystals (1‰ from ICPMS) involved loading 210 mg (0.572 mmol) of lead(II) bromide ($PbBr_2$, purity 98%), 50 mg of manganese(II) chloride ($MnCl_2$, purity 99.999%), 20 mL of octadecene, 2 mL of oleylamine (purity 98%), 2 mL of oleic acid, and 2 mL of trioctylphosphine (purity 97%) into a 100-mL three-neck flask. This was dried at 130°C for 45 min and heated to 150°C under vacuum. The yielded solution was then heated to 165°C under N_2 protection, after which 1.7 mL of pre-heated Cs-oleate precursors was rapidly injected into the solution. After having reacted for 10 s, a crude product was cooled to room temperature in an ice/water bath. Synthesis of high Mn^{2+}-doped perovskite nanocrystals (1.9‰ and 2.5‰ nomenclature) was the same as for the low-doped synthesis, except more $MnCl_2$ was used (70 mg and 80 mg of $MnCl_2$ precursor, respectively). We found that these materials are rather stable compared to non-perovskite nanocrystals. We have stored our samples in cuvettes with PTFE caps in air over the course of six months and found no significant changes to the PLQE over this period, indicating no alteration to the nanostructures due to aggregation or other harmful reactions resulting from an inherent material instability.

Structural characterization

The morphologies of the perovskite nanocrystals were characterized by a JEOL ARM 200F scanning-transmission electron microscope (STEM, 80 kV). Very similar nanocrystal size distributions centred around an average size of ~ 12 nm were found for undoped and doped samples alike. Thus, given that we also use the same stoichiometric amounts of precursors for the synthesis of undoped and doped materials, we can infer that we have very similar nanocrystal concentrations in undoped and doped solutions. The white spots found in Fig. 1a indicate electron-induced reduction of lead ions to metallic lead and are independent of the doping of the material. A detailed investigation of this phenomenon is described in ref. [2].

X-ray diffraction (XRD) data was acquired by a Bruker D2 Phaser.

Steady-state absorption

A Shimadzu UV-3600 Plus spectrophotometer was used to collect the steady-state absorbance spectra of samples, which uses a photomultiplier tube. The final data shown is corrected for by measuring the same cuvette with the solvent (toluene) only and subtracting this spectrum from the one with nanocrystals.

Steady-state and time-resolved photoluminescence (PL)

Steady-state and time-resolved PL spectra were recorded by a gated intensified CCD camera (Andor Star DH740 CCI-010) connected to a grating spectrometer (Andor SR303i). The pulsed output from a mode-locked Ti:Sapphire optical amplifier (Spectra-Physics Solstice, 1.55 eV photon energy, 80 fs pulse width, 1 kHz repetition rate) was used to produce 400 nm excitation via second harmonic generation in a β-barium borate crystal. The iCCD gate (width 2 ns) was electronically stepped in 2 ns increments, relative to the pump pulse, to enable ns-temporal resolution of the PL decay.

Photoluminescence quantum efficiency (PLQE)

PLQE data was collected using the method described by de Mello *et al.* [3]. Briefly, samples were positioned in an integrating sphere and excited at 400 nm, while the PL was collected with an Andor Shamrock spectrometer and Andor iDus CCD array. A corrected value is then determined by collecting the light from the sphere without a sample, without hitting the sample and with hitting the sample, respectively. Stated values were determined on triplicate samples which were each measured twice, hence reporting the average of six measurements for each composition.

Transient absorption (TA) spectroscopy

TA is a form of pump-probe spectroscopy which measures the spectrally resolved variation in absorption by a sample under photoexcitation by a pump source. By varying the pump-probe time delay, the carrier recombination kinetics of the sample can be investigated. The third harmonic of a pulsed Nd:YVO_4 laser (Picolo-AOT MoPa) was used as the pump beam (1 ns pulse width, 500 Hz repetition rate, 355 nm) for the ns regime measurements. The probe spectrum was generated using a white light quasi-continuum generated through pumping a CaF_2 window with the 800 nm fundamental of a Ti:Sapphire amplifier (Spectra-Physics Solstice). A delay generator was used to electronically vary the pump-probe delay. For the short time fs-regime, the pump beam was the second harmonic (400 nm) generated by the 800 nm fundamental passing through a β-barium borate crystal. Transmitted probe and reference pulses were recorded with an NMOS linear image sensor (Hamamatsu S8381-1024Q) and processed by a customized PCI interface from Entwicklungsbüro Stresing.

Calculation of exciton recombination rates

We determine first the total exciton decay lifetime from time-resolved PL (see Fig. S2). We found that the PL kinetics for all compositions can only be fitted to a high satisfying level if not less than the sum of three exponentials is used according to:

$$PL(t) = \sum_{i=1}^{n} a_i e^{-t/\tau_X^i} \quad \text{(S1)}$$

with τ_X^i as effective single-exciton lifetime (including both radiative and nonradiative contributions) and a_i the relative fraction (in sum being 1) of NCs in the i-th out of n sub-ensembles possessing this lifetime. The necessity of a triexponential fit is in line with other reports on similar perovskite nanocrystals, for example by the groups of Klimov [4] or Herz [5]. As described in the references, we assume all nanocrystals measured in the ensemble to possess the same radiative recombination constant $k_{r,X}$, which is the inverse of the intrinsic radiative lifetime$\langle\tau_{r,X}\rangle$, while the nonradiative recombination constant $k_{nr,X}$ can differ for sub-ensembles, e.g. due to different trap densities or identities. From weighting the individual sub-ensemble lifetimes via a_i, we can determine the average PL lifetime $\langle\tau_X\rangle$ and hence the average total recombination constant $\langle k_X\rangle = \langle\tau_X\rangle^{-1}$. The PLQE of each sub-ensemble is the ratio of its radiative to total recombination rate, and the total PLQE of the ensemble is therefore:

$$PLQE = \frac{\langle\tau_X\rangle}{\langle\tau_r\rangle} \quad \text{(S2)}$$

From measuring PLQE and total PL lifetimes, the intrinsic radiative lifetime and hence recombination constant can be determined. Furthermore, the average non-radiative recombination rate can be calculated, since

$$k_{nr,X} = \langle k_X\rangle - k_{r,X} \quad \text{(S3)}$$

The composition-dependent constants are reported in Supplementary Table T1. Our extracted radiative recombination constants for the materials match well the kinetics of the initial few ns, and [together with the arguments from above in comments 5) and 6)] are indicative of radiative recombination dominating early time delays. The subsequent PL drop in the intermediate and late time delays are then mostly non-radiative contributions, modelled by the multi-exponential fit to cover the ensemble's distribution of individual non-radiative decay rates, which in sum yield the averaged non-radiative rate constant reported in Supplementary Table T1.

Calculation of average excitations per nanocrystal $\langle N\rangle$ via two independent approaches

1. <u>Estimation based on bulk-estimate depending on NC concentration and steady-state absorbance</u>

The excitation fluence per pulse F_{ex} (in μJ cm^{-2}) is related to the laser power P used for photoexcitation of the samples in solution, the repetition rate R_{rep} and the beam spot radius r_{ex} through:

$$F_{ex} = \frac{P}{R_{rep}\pi r_{ex}^2} \quad \text{(S4)}$$

The photon fluence per pulse j (in cm^{-2}) is given by:

$$j = \frac{P\lambda\pi r_{ex}^2}{R_{rep}hc} \quad \text{(S5)}$$

where λ is the excitation wavelength, c is the speed of light in a vacuum, and h is Planck's constant. The absorbed photon density per pulse j_{abs} (in cm^{-3}) in the cuvette of pathlength l that is being absorbed can be inferred from measuring the UVvis absorbance A at the excitation wavelength and reads:

$$j_{abs} = j\frac{A}{l} \quad \text{(S6)}$$

The density of NCs in solution ρ_{NC} (in cm^{-3}) is related to the NC concentration c_{NC} (in mg mL^{-1}) and the volume of a NC V_{NC} and the weight density of the material ρ_m via:

$$\rho_{NC} = \frac{c_{NC}}{V_{NC}\rho_{NC}} \quad \text{(S7)}$$

Therefore, the average excitations per NC $\langle N\rangle$ determined by this approach is:

$$\langle N\rangle = j_{abs}/\rho_{NC} \quad \text{(S8)}$$

2. <u>Estimation based on Poisson distribution of initial excitations in the NC ensemble</u>
Under the general assumption of Poisson statistics of photon absorption events, the initial occupancy of photo-induced excitations in a NC ensemble follows a Poisson distribution [6]. Thus, the probability p of a NC to contain i excitations at early times after excitation is:

$$p_i = \langle N \rangle^i / i! \times e^{-\langle N \rangle} \tag{S9}$$

After multi-exciton decay is completed, which we confirm is the case after few ps by performing ultrafast TA spectroscopy further below, for the ns-TA data discussed in the main text and Fig. 3, only up to one excitation can be left on a single NC. By measuring the (long-time) TA intensity I_{TA} as a function of photon fluence j, set out in Eq. S5 above, we can extract the average excitations per NC $\langle N \rangle$ from the following saturation function by fitting the data:

$$I_{TA} \sim 1 - p_0 = 1 - e^{-\langle N \rangle} \tag{S10}$$

Both approaches yielded very similar results for $\langle N \rangle$ as a function of excitation fluence in our experiments.

Calculation of wavefunction overlap of electrons and holes
The wavefunction overlap Θ of electrons and holes is defined as:

$$\Theta = |\int \psi_e^* (r) \psi_h(r) dV|^2 \tag{S11}$$

Following an approach previously used for example by the groups of de Mello Donegá [7,8], or Kelley [9,10] and originally described by Efros and Rodina [11], the overlap integral is directly related to the oscillator strength and radiative rate via:

$$k_{r,X} = \frac{2e^4 n}{\pi \varepsilon_0 m_0^3 c^3} |F|^2 \frac{m_0^2 E_g E_p \Theta}{3e^2 \hbar^2} \tag{S12}$$

where e is the elementary charge, n the refractive index, ε_0 is the vacuum permittivity, c is the speed of light in a vacuum, m_0 is the free electron rest mass, $\hbar$ is the reduced Planck's constant, and E_g is the energy of the optical transition.
$F = 3\varepsilon_m / (\varepsilon_s + 2\varepsilon_m)$ is the local field factor to account for the screening of the nanoparticle, assuming a random orientation of NCs with respect to the electric field of the interacting light. For simplicity, we modelled the NCs as spheres, with ε_m and ε_s being the dielectric constants of the medium and semiconductor, respectively. A more rigorous treatment of the electric field strength inside cubic nanocrystals can be found in ref. [12] Importantly, this will not influence the changes to the wavefunction overlap, since the same shape of NCs is maintained without and with doping, as checked with TEM. E_p is the Kane energy, usually found for III-V semiconductors to be on the order of 20 eV [4], but notably very close to 40 eV for $CsPbX_3$ perovskites due to the different orbital contributions to the valence and conduction band edges here, independent of halide choice [12].
The formal derivation of Eq. S12 can be found in the early works by Efros [11,13], and later also discussed together with Bawendi [14]. Importantly, we find that the measured increases in oscillator strength (from both TA and Tr-PL/PLQE) and thus also radiative rate *cannot* be explained by the only slight increase in bandgap and Kane energy upon Mn doping. This is because the doping level in the permille-regime is too small to significantly alter the global properties of the semiconductor, while still an exciton will experience the presence of a dopant, given that the distance between the distributed Mn ions is less than 5 nm and the exciton Bohr radius already approx. 2.5 nm (then followed by exciton diffusion as well). Thus, the overlap integral increases significantly in order to account for the enhancement observed experimentally. This stronger overlap is a direct consequence of the lattice-periodicity breaking by the manganese dopants which leads to the localization of charges at these sites, thereby increasing the radiative rate of excitons in their vicinity.

First principles calculations

Computational methods and parameters

We use first principles methods based on density functional theory [15,16] combined with the projector-augmented wave method (PAW) [17,18] as it is available in the Vienna *ab initio* simulation package (VASP) [19,20]. We use two different exchange correlation functionals, namely, the semilocal generalized gradient approximation of Perdew-Burke-Ernzerhof (PBE) revised for solids (PBEsol) [21,22] and the hybrid Heyd-Scuseria-Ernzerhof functional (HSE06) [23,24], albeit the latter is only used as a benchmark as well as for the 3.7 % Mn-doped $CsPbCl_3$ (Fig. 3c and d) due to a large computational expense. We employ PAW potentials containing nine valence electrons for Cs ($5s^2 5p^6 6s^1$), fourteen valence electrons for Pb ($5d^{10} 6s^2 6p^2$), seven valence electrons for Cl ($3s^2 3p^5$) and Br ($4s^2 4p^5$), as well as thirteen valence electrons for Mn ($3p^6 4s^2 3d^5$). Based on convergence tests we use a plane-wave basis set with an energy cutoff of 500 eV (pristine unit cells) and 250 eV (doped supercells) with a Brillouin-zone grid of 6×6×6 Γ-centered k-points for the structural relaxation of the unit cell. We relax the cubic primitive cell for the single halide perovskites using the conjugate gradient algorithm until the energy differences are converged within 10^{-6} eV, with a Hellman-Feyman force convergence threshold of 0.01 eV/Å.

For the single halide Mn-doped $CsPbCl_3$ and $CsPbBr_3$ we take the relaxed cubic primitive cells as reference structures to construct 2×2×2 and 3×3×3 supercells with uniform 3×3×3 and 2×2×2 Γ-centered k-point grids, respectively. We replace a B-site Pb with a Mn and relax the structure using the conjugate gradient algorithm as stated above. For the mixed-halide systems we use a configuration below generated *via* the special quasi-random structure (SQS) method [25] and again replace a Pb atom with a Mn atom. Using DFT+U theory [26] we further test the dependence of the lattice parameters on different Hubbard $U_{eff} = U - J$ parameters and spin configurations within the Dudarev *et al.* formalism [27] as it is implemented in VASP [28,29]. A more detailed description for the geometry optimizations is found below.

Benchmark of U+J parameters for different ground-state spin configurations

A known drawback of DFT is the poor treatment of strongly correlated systems. GGA+U is one of the simplest approaches that was formulated to improve the description of the ground state of correlated systems and that is frequently used to circumvent the issue of predicting the magnetic ground state with a small computational expense [30]. For different U_{eff} we optimize the geometry for different spin configurations and find that the lattice parameters for nonmagnetic spin configurations decreases by ~1% with respect to the ferromagnetic spin alignment. The change in the lattice parameters for different U_{eff} remains negligible. For all U_{eff} there was no preferred spin-configuration, suggesting that the ground state for a 3×3×3 supercell doped with a Mn atom (3.7%) has no preferred magnetic order. These observations hold true for all systems. The final structures are relaxed with a *U*-parameter of value 4 and a *J*-parameter of value 0.5 ($U_{eff} = 3.5$).

Benchmark of U+J parameters for correct band ordering in doped supercells

Another issue of DFT is the over-delocalization of *d*-states which falsely leads to over-hybridization with the band edges of the system. This error can also be avoided by incorporating a *U* and *J* correction into the exchange correlation functional. Within the GGA+U formalism, the electrons of the system are separated into two subsystems. The first subsystem includes delocalized *s* and *p*-states, that can be described with the GGA. The second subset entails *d* electrons that require an additional on-site screened Coulomb potential. This Coulomb potential acts on the subset of *d* electrons and localizes the orbitals, mimicking the *d-d* repulsion. In contrast to that approach, hybrid functionals incorporate a portion of exact exchange in form of a Fock operator that acts on all Kohn-Sham states [30].

Albeit hybrid functionals have established themselves as the more accurate method, we do not use them for all systems because they are associated with a large computational cost. Therefore, we calculate the projected density of states for the Mn-doped 2×2×2 supercells of $CsPbCl_3$ and $CsPbBr_3$ using PBEsol with different *U* and *J* parameters as well as using the HSE06 functional with an energy cut-off of 250 eV and a 3×3×3 Γ-centered k-point grid. We do not use the results for microscopic interpretations as we assume the doping concentrations to be too large to provide a realistic model system for low-concentration doping. We find that a *U*-parameter of 4 and a *J* -parameter of 0.5 ($U_{eff} = 3.5$) resemble

the projected density of states obtained via the HSE06 functional the most. As expected, using HSE06 increases the band gap for about 1 eV, while the band ordering is correctly captured by the U_{eff} = 3.5 value.

Hereafter we calculate the band structure and the projected density of states for the 3.7% doped $CsPbBr_3$, $CsPb(Cl,Br)_3$ and $CsPbBr_3$ using the DFT + U_{eff} = 3.5 method with a 2×2×2 Γ-centered k-point grid. The projected density of states and folded band structures are depicted in Fig. S7 and Fig. S8, respectively. While $CsPbBr_3$ and $CsPb(Cl,Br)_3$ prove particularly robust with respect to the chosen Hubbard U parameters, the electronic structure of 3.7% doped $CsPbCl_3$ appears much more sensitive due to the critical in-gap positions of Mn 3*d* states and the somewhat poor description of Mn 4*s* states. Therefore, we also calculate the projected density of states at the HSE06 level of theory which is illustrated in the manuscript (Fig. 3d).

Spin-orbit coupling is included throughout all electronic structure calculations using the second-variational method, in which the spin-orbit interaction is included perturbatively to the scalar-relativistic Hamiltonian [31].

Supplementary Data

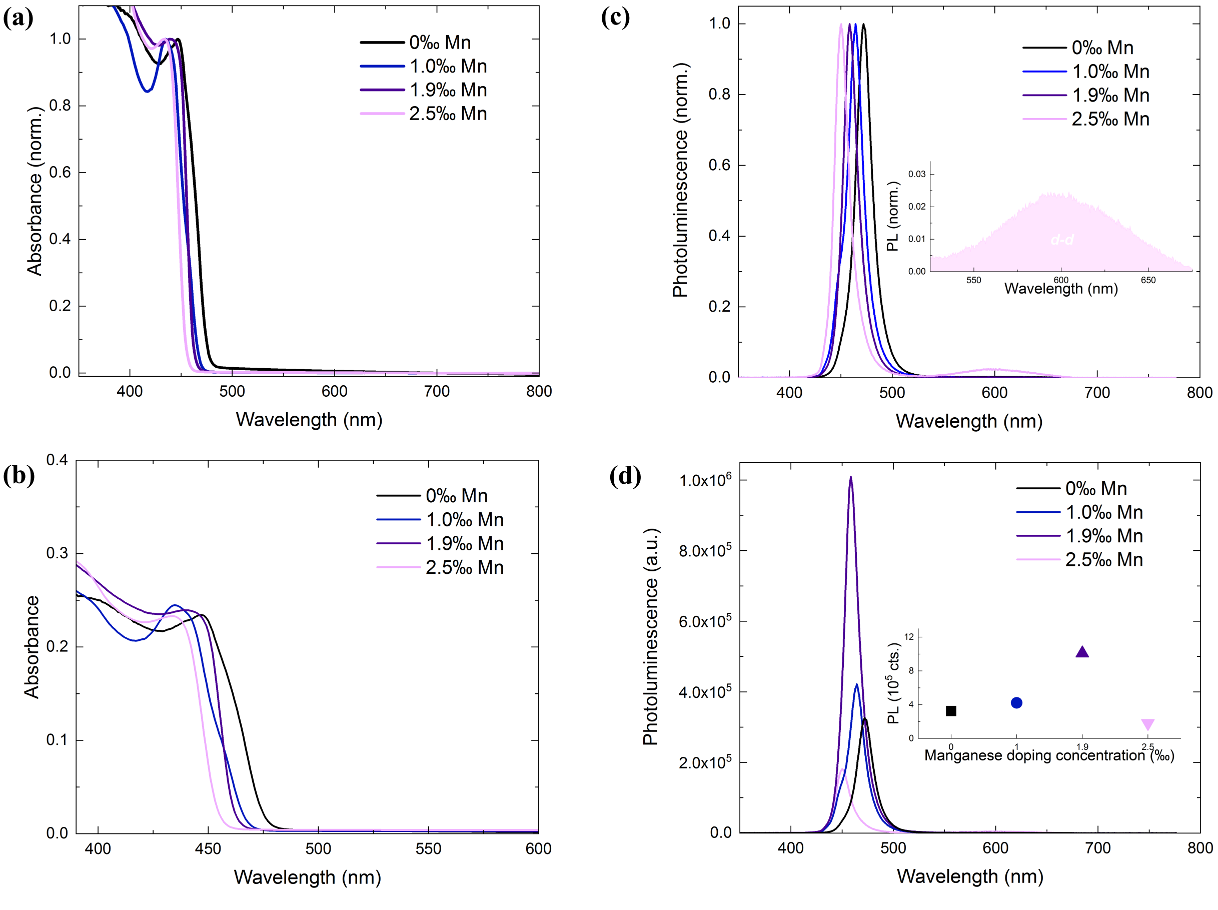

Supplementary Figure S1. Full range steady-state absorbance and photoluminescence spectra. (a) Normalized and (b) absolute absorbance spectra showing the absence of absorption of the spin-forbidden manganese *d-d* transition. (c) Normalized PL spectra, showing manganese emission only present in the highest doping level of 2.5‰ Mn (inset: zoom-in of the broad *d-d* emission). (d) Absolute PL spectra (inset: PL maxima as a function of doping level with the strongest PL achieved at 1.9‰ Mn).

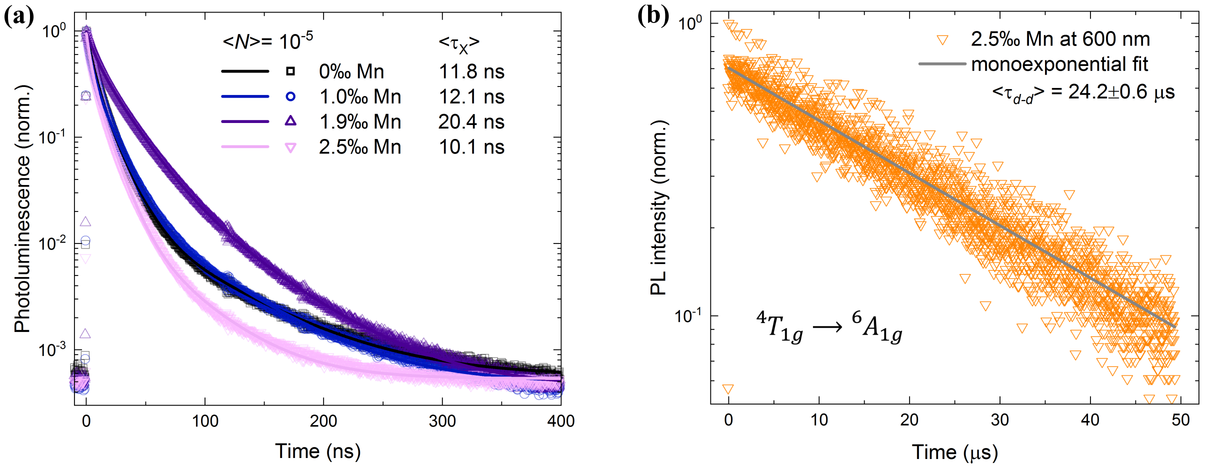

Supplementary Figure S2. Enhanced excitonic emission through manganese doping. (a) Time-resolved photoluminescence decay of the excitonic emission at approximately 10^{-5} excitations per nanocrystal. Triexponential fits yield the highe st average PL lifetime $\langle \tau_X \rangle$ for the 1.9‰ doping level. (b) Long-lived manganese emission from the sample with highest doping level. We note that the extracted lifetime is underestimated, since it was not possible to measure with longer time delays than 50 µs at our setup. All measurements taken at 400 nm excitation using time-correlated single-photon counting (TCSPC).

Mn-doping concentration (atom‰)	$\langle k_X \rangle$ (10^7 s^{-1})[a] [measured]	PLQE (%)[a] [measured]	$k_{r,X}$ (10^7 s^{-1}) [calculated]	$k_{nr,X}$ (10^7 s^{-1}) [calculated]
0	8.48	17.71	1.50	6.99
1.0	8.26	21.66	1.79	6.49
1.9	4.90	38.40	1.88	3.02
2.5	9.90	25.02	2.48	7.41

[a]Measurements taken at 400 nm excitation at a low-energy flux of 127 µW cm^{-2}.

Supplementary Table T1. Manganese-doping simultaneously enhances radiative and decreases non-radiative recombination. The inverse of the average PL lifetime as determined from TCPSC (Fig. S2), $\langle k_X \rangle$, and the non-radiative recombination constant, $k_{nr,X}$, are minimal for the 1.9‰ doping level, while the PLQE is here maximal. The radiative recombination constant, $k_{r,X}$, continues to further increase with higher doping concentration. $k_{r,X}$ was calculated according to $k_{r,X} = \mathrm{PLQE} \times \langle k_X \rangle$ and $k_{nr,X}$ was calculated according to $k_{nr,X} = \langle k_X \rangle - k_{r,X}$.

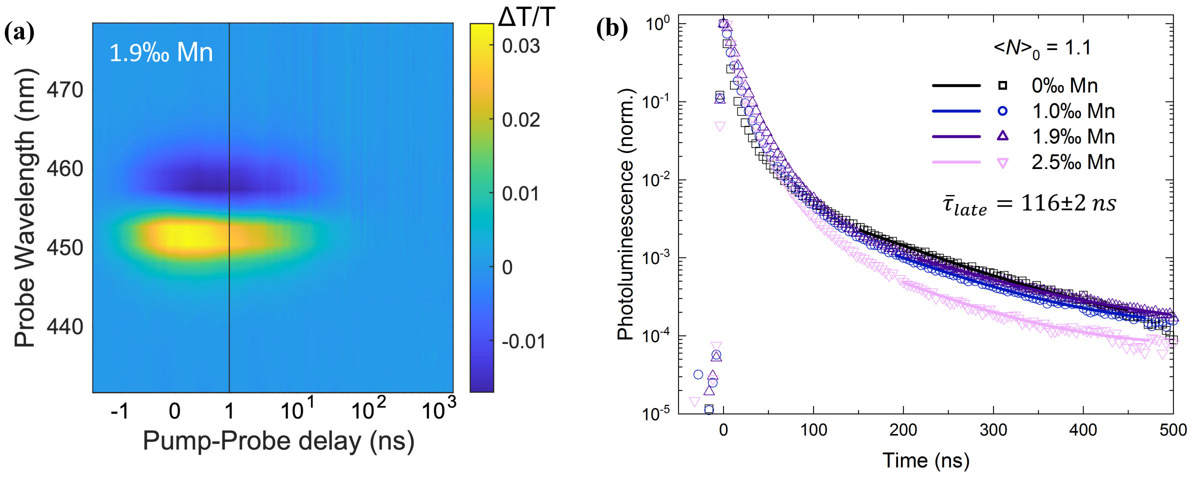

Supplementary Figure S3. Full TA data and late-time PL tail fits. (a) 2D pseudo-colour plot of exemplary long-time TA data, here shown for the 1.9‰ doping level at approx. 1.1 initial average excitations per NC. (b) PL kinetics with individual fits shown. At sufficiently large time delays (approx. 200 ns) after photoexcitation with a 400 nm pulse, all compositions exhibit a similar monoexponential decay which we attribute to trap-limited Shockley-Read-Hall recombination. The average lifetime of this late-time component is determined through a global fit where all data share one lifetime as parameter. This lifetime is 116±2 ns and stated in the main text, indicating similar trap-densities independent of doping level.

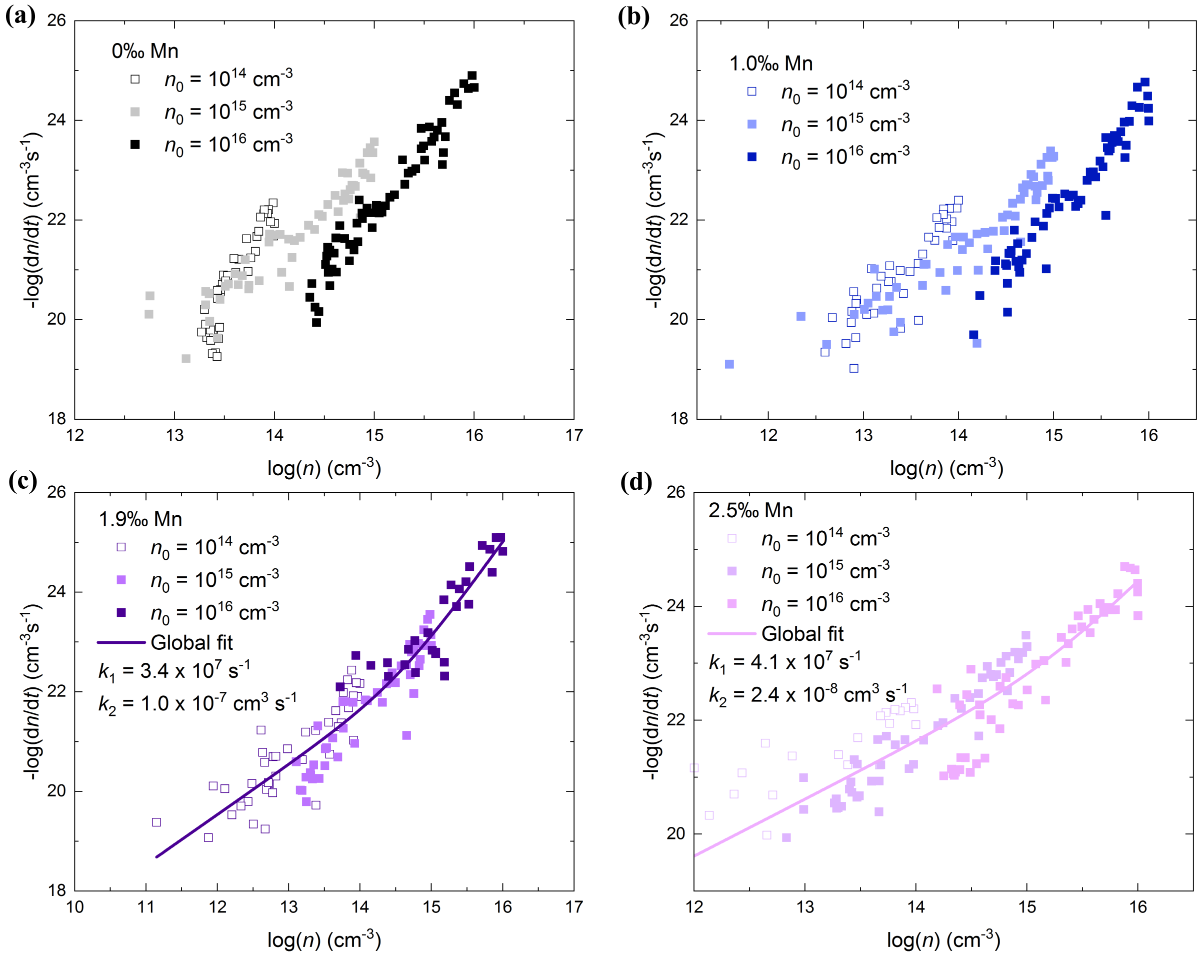

Suppelementary Figure S4. Change of charge carrier population rate as function of carrier density for different doping-levels. (a)-(d) Changes for different Mn doping levels, determined from TA ground-state bleach kinetics at difference excitation densities, indicated by open, light and dark filled symbols, respectively. Manganese doping reduces the fast non-radiative loss channels, thereby removing the history dependence on initial carrier density at sufficient doping levels in (c) and (d).

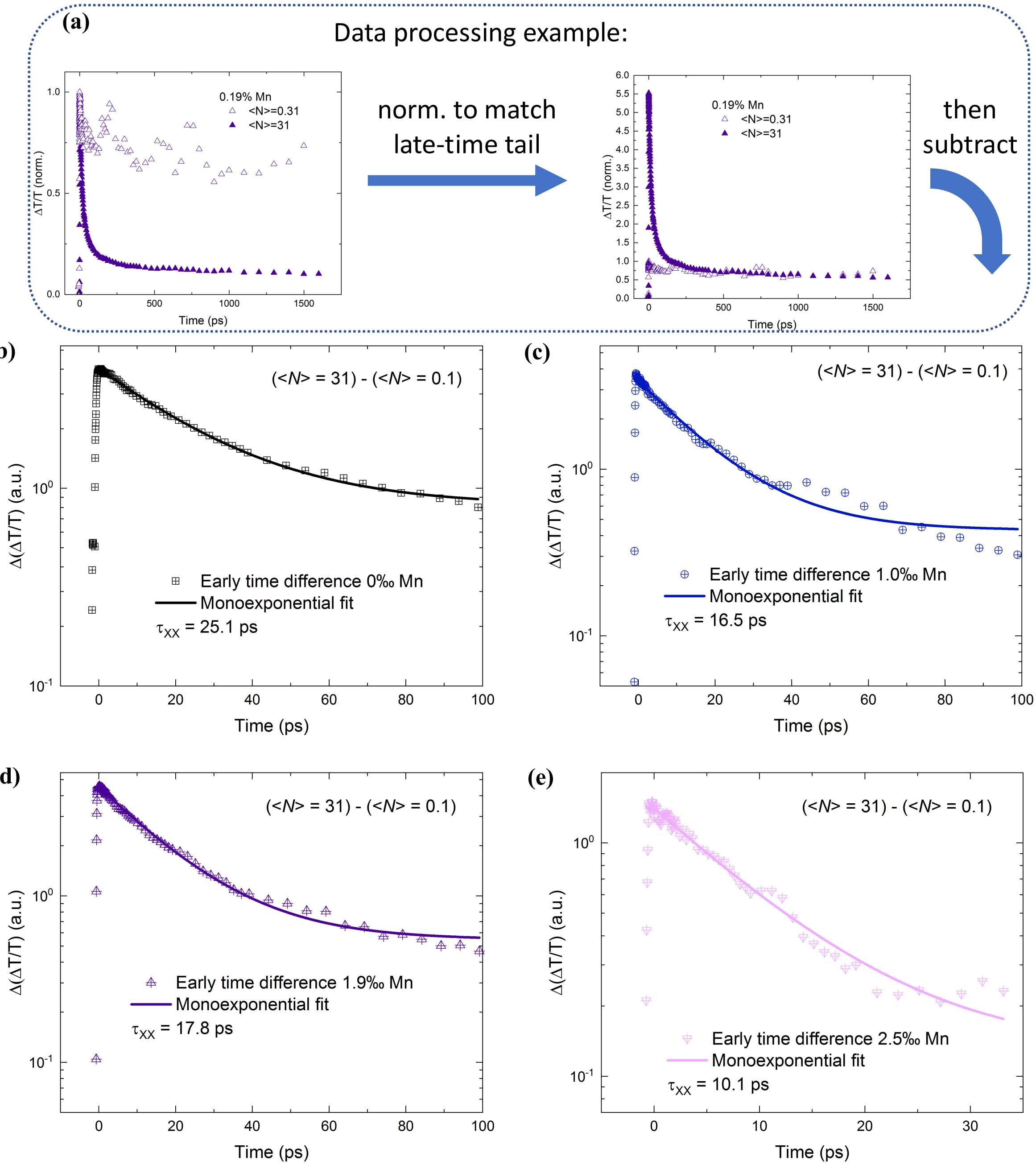

Supplementary Figure S5. Biexciton lifetimes extracted from fluence-dependent TA. (a) Example of procedure to extract biexciton lifetime: For a given composition, a low-fluence TA kinetics, where there is no significant biexciton contribution present, as well as a high-fluence kinetics is recorded. Then both are normalized to match their late-time single-exciton decay and subtracted from each other. (b)-(e) Monoexponential fits to the biexciton decay component for different doping levels, indicating an overall faster biexciton decay with increasing doping level, ranging from 25 to 10 ps lifetime.

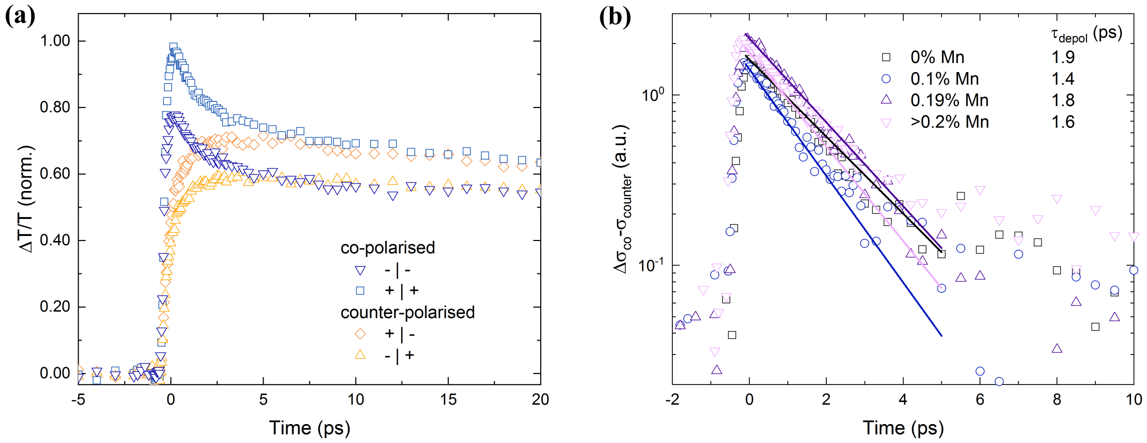

Supplementary Figure S6. Depolarization dynamics of ground-state bleach signal from circularly-polarized TA. For this experiment, the pump and the probe beams were circularly-polarized through sets of linear polarizers and quarter-waveplates each. (a) Representative co- and counter polarized configurations for the 1.9‰ doping-level composition, indicating a prompt decay for the co- and a delayed decay for the counter-configuration, respectively. The kinetics are normalized to their late-time decays, respectively. (b) Subtraction of the normalized kinetics in (a) yields depolarization lifetimes ranging from 1.4 to 1.9 ps with no significant influence of Mn-doping. The average depolarization lifetime of 1.6 ps is reported in the main text. We note that this lifetime is shorter than previously published results using this technique on bulk $MaPbI_3$ material of about 7 ps [32]. This can be explained by the smaller exciton binding energy in their material compared to our nanocrystals, as well as the temperature-difference, since they conduct measurements at ca. 77 K, while we measured at room temperature, hence potentially reducing depolarisation lifetimes by enhanced phonon scattering.

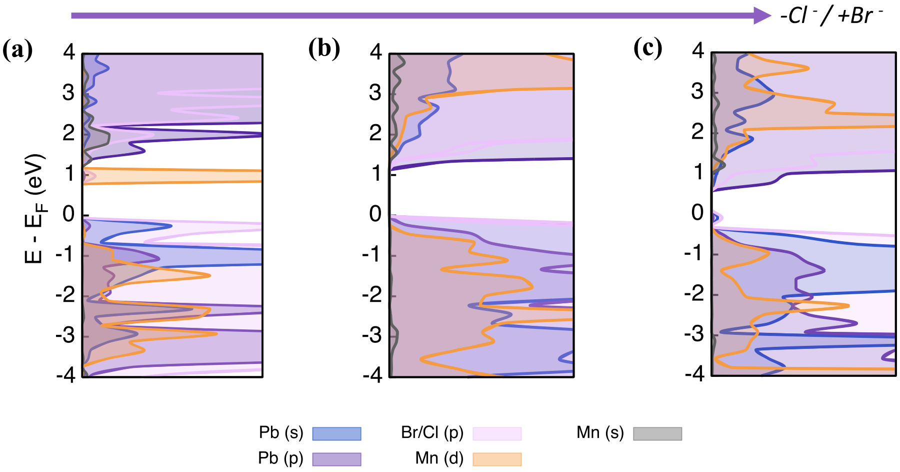

Supplementary Figure S7. Projected density of states of the test systems calculated with PBEsol+U (U_{eff} = 3.5). We calculate the projected density of states for 3.7 % Mn-doped (a) $CsPbCl_3$, (b) $CsPb(Cl,Br)_3$ and (c) $CsPbBr_3$ and observe following trends: With increasing bromide content the band gap narrows, which is in accordance to the observation expected in the manganese-free cases. Another feature we find is that, with increasing bromide content, the lowest lying unoccupied Mn 4*s* orbitals energetically mostly remain in the same energy regime (1 eV–1.5 eV). In combination with the band gap narrowing, this suggests that hybridization with the 4*s* states is reduced when we move from the chloride to the bromide. In addition, we observe in (a) a large contribution of Mn 4*s* states at the conduction band edge. We further investigate the role of the Mn 4*s* states using HSE06 (Fig. 3d) and deduce that the Mn-doped $CsPbCl_3$ requires exact exchange not only to get a correct representation of the 3*d* states but also of the 4*s* states in the low dopant-concentration regime.

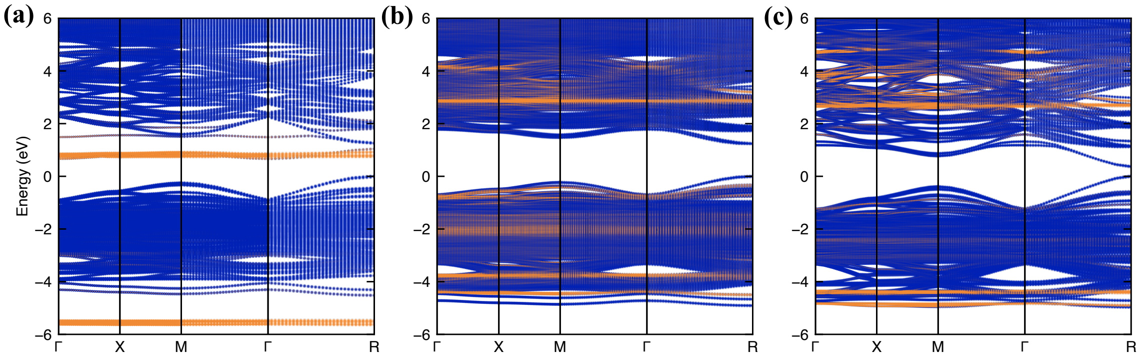

Supplementary Figure S8. Folded band structures of the test systems calculated with GGA+U (*U_{eff}* = 3.5). The assigned high-symmetry paths correspond to the path of the pristine systems. The Mn 3*d* contributions are highlighted in orange. (a) shows the folded band structure of Mn-doped $CsPbCl_3$ with a nominal doping concentration of 3.7%. The most prominent features are the flat band edges of the perovskite and the Mn 3*d* states within the band gap. For (b), which represents the band structure of Mn-doped $CsPb(Cl,Br)_3$ the 3*d* orbitals are shifted towards the lower valence bands and the higher conductions bands. The same holds true for (c), which shows the band structure of doped $CsPbBr_3$. Here we find more disperse band edges compared to (a) and (b), again with no 3*d* contributions in proximity to the band. The tuneable positions of the Mn 3*d* states as well as the changing dispersiveness of the band edges demonstrate the importance of the halide composition in Mn-doped metal halide perovskites.

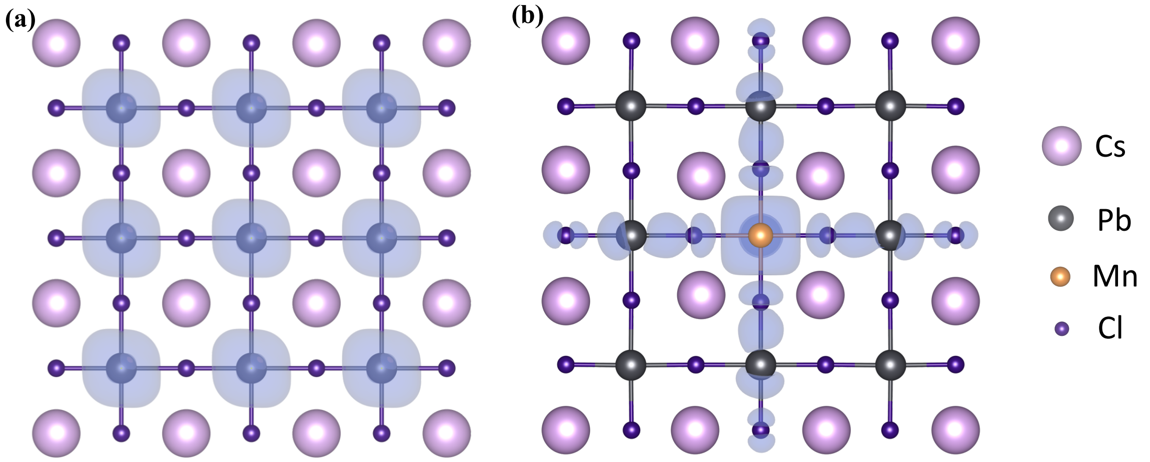

Supplementary Figure S9. Real space charge distribution of the conduction band minimum that is located at the high-symmetry point *R* (0.5, 0.5, 0.5). (a) Shows the charge distribution at the conduction band minimum of pristine $CsPbCl_3$ with a charge density of 1.6×10^{-3} *e* Å^{-3}. The conduction band minimum here is generated by a hybridization of unoccupied Pb 6*p* orbitals that are fully delocalized over all Pb centers. (b) Shows the charge density localization of 3.7 % Mn-doped $CsPbCl_3$ with a charge density of 2.36×10^{-3} *e* Å^{-3}. The conduction band minimum now significantly mixes with the Mn 4*s* orbital which is energetically aligned with the Pb 6*p* states. Additional hybridization with the 3*p* orbitals of the complexing Cl atoms leads to an isotropically localized state around the guest atom.

Effective mass approximation

System	Pristine	Mn-doped	m_h gain doped/undoped (%)	Pristine	Mn-doped	m_e gain doped/undoped (%)
	$m_h(m_0)$			$m_e(m_0)$		
$CsPbBr_3$	0.161	0.186	116	0.165	0.199	121
$CsPb(Br,Cl)_3$	0.169	0.319	189	0.180	0.387	215
$CsPbCl_3$	0.193	0.316	164	0.206	0.596	289

Supplementary Table T2: Effective mass approximation confirms doping-induced carrier localisation. Effective electron and hole masses for the pristine and the 3.7% Mn-doped model systems are shown, respectively. The curvature effective masses were directly calculated from the electronic band structures *via* a parabolic fit ($R \rightarrow \Gamma$) of the electronic dispersion $\frac{1}{m_c} = \frac{1}{\hbar^2}\frac{\partial^2 E}{\partial k^2}$. It should be noted, though, that the effective mass approximation (especially conducted using a parabolic fit of the energy dispersion) is quite a crude approximation and sensitive to the DFT+U(+J) parameters. The sensitivity with respect to the chosen parameters is more prominent when Mn states mix with the band edges. There are multiple studies showing that non-parabolicity of the bands can change the effective masses significantly. Further details are denoted in the reference [33]. Thus, the absolute values calculated here should only be taken as a qualitative measure, confirming the experimentally observed increases in oscillator strength and concomitant radiative rates.

Discussion of Mn-doped perovskites with different halide compositions

We note that the hybridization of the Mn 4*s* orbitals with the host shown here for the Mn-doped pure-chloride composition is halide-dependent (see Fig. S7): With increasing Br concentration the unoccupied perovskite states shift towards lower energies, while the Mn 4*s* states remain invariant. This off-set of the relative energies causes a reduced hybridization. The resulting loss in localization could explain why similarly beneficial doping effects on the radiative rate of the perovskite NCs have not been reported in the literature for pure-bromide compositions so far.

Further, we observe that the relative position of the vacant Mn 3*d* states is not only dependent on the Mn concentration but on the halide content, too: With increasing Br content, the unoccupied Mn 3*d* orbitals are pushed towards higher energies, such that *d-d* transitions that were previously lying within the host band gap become inaccessible as an alternative decay pathway. This suggests that a balanced halide ratio and distribution is beneficial in order to suppress this host exciton decay channel. We conclude that both the Mn *and* the halide concentration are levers that can be used to tune localization and thus PLQE. This further points towards $MnCl_2$ acting not only as a means to fill halide vacancies and reduce trap densities that cause non-radiative decay, but more importantly to engineer the exciton wave function for optimized radiative yields through the halide ratio and distribution of Mn centers.

A recent study on Mn doping in bulk methylammonium lead iodide reports similarly on increased effective masses upon doping, though in their case the hole curvature flattens more strongly [34]. This could be a further indication of the role of the halide ion (purely iodide in their case) and its ability to hybridize with the Mn orbitals to generate new bands with favorable energy levels and curvature (*i.e.* localization).

References

[1] S. Hou, M. K. Gangishetty, Q. Quan, and D. N. Congreve, Joule **2**, 2421 (2018).
[2] Z. Dang, J. Shamsi, F. Palazon, M. Imran, Q. A. Akkerman, S. Park, G. Bertoni, M. Prato, R. Brescia, and L. Manna, ACS Nano **11**, 2124 (2017).
[3] J. C. de Mello, H. F. Wittmann, and R. H. Friend, Adv. Mater. **9**, 230 (1997).
[4] N. S. Makarov, S. Guo, O. Isaienko, W. Liu, I. Robel, and V. I. Klimov, Nano Lett. **16**, 2349 (2016).
[5] S. G. Motti, F. Krieg, A. J. Ramadan, J. B. Patel, H. J. Snaith, M. V. Kovalenko, M. B. Johnston, and L. M. Herz, Adv. Funct. Mater. **1909904**, 1 (2020).
[6] V. I. Klimov, J. Phys. Chem. B **104**, 6112 (2000).
[7] C. D. M. Donegá and R. Koole, J. Phys. Chem. C **113**, 6511 (2009).
[8] A. Granados Del Águila, E. Groeneveld, J. C. Maan, C. De Mello Donegá, and P. C. M. Christianen, ACS Nano **10**, 4102 (2016).
[9] K. Gong, J. E. Martin, L. E. Shea-Rohwer, P. Lu, and D. F. Kelley, J. Phys. Chem. C **119**, 2231 (2015).
[10] D. P. Morgan and D. F. Kelley, J. Phys. Chem. C **123**, 18665 (2019).
[11] A. L. Efros and A. V Rodina, Phys. Rev. B **47**, 10005 (1993).
[12] M. A. Becker, R. Vaxenburg, G. Nedelcu, P. C. Sercel, A. Shabaev, M. J. Mehl, J. G. Michopoulos, S. G. Lambrakos, N. Bernstein, J. L. Lyons, T. Stöferle, R. F. Mahrt, M. V. Kovalenko, D. J. Norris, G. Rainò, and A. L. Efros, Nature **553**, 189 (2018).
[13] A. L. Efros, Phys. Rev. B **46**, 7448 (1992).
[14] A. L. Efros, M. Rosen, M. Kuno, M. Nirmal, D. Norris, and M. Bawendi, Phys. Rev. B - Condens. Matter Mater. Phys. **54**, 4843 (1996).
[15] P. Hohenberg and W. Kohn, Phys. Rev. **136**, B864 (1964).
[16] W. Kohn and L. J. Sham, Phys. Rev. **140**, A1133 (1965).
[17] P. E. Blöchl, Phys. Rev. B **50**, 17953 (1994).
[18] G. Kresse and D. Joubert, Phys. Rev. B **59**, 1758 (1999).
[19] G. Kresse and J. Furthmüller, Phys. Rev. B **54**, 11169 (1996).
[20] G. Kresse and J. Furthmüller, Comput. Mater. Sci. **6**, 15 (1996).
[21] J. P. Perdew, A. Ruzsinszky, G. I. Csonka, O. A. Vydrov, G. E. Scuseria, L. A. Constantin, X. Zhou, and K. Burke, Phys. Rev. Lett. **100**, 136406 (2008).
[22] J. P. Perdew, A. Ruzsinszky, G. I. Csonka, O. A. Vydrov, G. E. Scuseria, L. A. Constantin, X. Zhou, and K. Burke, Phys. Rev. Lett. **102**, 39902 (2009).
[23] J. Paier, M. Marsman, K. Hummer, G. Kresse, I. C. Gerber, and J. G. Ángyán, J. Chem. Phys. **124**, 154709 (2006).
[24] J. Paier, M. Marsman, K. Hummer, G. Kresse, I. C. Gerber, and J. G. Ángyán, J. Chem. Phys. **125**, 249901 (2006).
[25] A. Zunger, S.-H. Wei, L. G. Ferreira, and J. E. Bernard, Phys. Rev. Lett. **65**, 353 (1990).
[26] V. I. Anisimov, I. V Solovyev, M. A. Korotin, M. T. Czy\ifmmode \dotz\else ż\fiyk, and G. A. Sawatzky, Phys. Rev. B **48**, 16929 (1993).
[27] S. L. Dudarev, G. A. Botton, S. Y. Savrasov, C. J. Humphreys, and A. P. Sutton, Phys. Rev. B **57**, 1505 (1998).
[28] O. Bengone, M. Alouani, P. Blöchl, and J. Hugel, Phys. Rev. B **62**, 16392 (2000).
[29] A. Rohrbach, J. Hafner, and G. Kresse, J. Phys. Condens. Matter **15**, 979 (2003).
[30] B. Himmetoglu, A. Floris, S. de Gironcoli, and M. Cococcioni, Int. J. Quantum Chem. **114**, 14 (2014).
[31] D. D. Koelling and B. N. Harmon, J. Phys. C Solid State Phys. **10**, 3107 (1977).
[32] D. Giovanni, H. Ma, J. Chua, M. Grätzel, R. Ramesh, S. Mhaisalkar, N. Mathews, and T. C. Sum, Nano Lett. **15**, 1553 (2015).
[33] L. D. Whalley, J. M. Frost, B. J. Morgan, and A. Walsh, Phys. Rev. B **99**, 1 (2019).
[34] D. Bartesaghi, A. Ray, J. Jiang, R. K. M. Bouwer, S. Tao, and T. J. Savenije, APL Mater. **6**, (2018).
